# Conducting a high-stakes OSCE in a COVID-19 environment

**DOI:** 10.15694/mep.2020.000054.1

**Published:** 2020-03-27

**Authors:** Katharine Boursicot, Sandra Kemp, Thun How Ong, Limin Wijaya, Sok Hong Goh, Kirsty Freeman, Ian Curran

**Affiliations:** 1 Duke-NUS Medical School; 2 Curtin Medical School; 3 Singapore General Hospital

**Keywords:** OSCE, Clinical Examination, Pandemic, COVID-19, Simulation, Clinical Skills Assessment, Performance Assessment, Quality Assurance

## Abstract

This article was migrated. The article was marked as recommended.

The COVID-19 pandemic has presented significant challenges for medical schools. It is critical to ensure final year medical school students are not delayed in their entry to the clinical workforce in times of healthcare crisis. However, proceeding with assessment to determine competency for graduation from medical school, and maintaining performance standards for graduating doctors is an unprecedented challenge under pandemic conditions. This challenge is hitherto uncharted territory for medical schools and there is scant guidance for medical educators. In early March 2020, Duke-National University Singapore Medical School embraced the challenge for ensuring competent final year medical students could complete their final year of studies and graduate on time, to enter the medical workforce in Singapore without delay. This paper provides details of how the final year clinical performance examinations were planned and conducted during the COVID-19 pandemic. The aim of the paper is to provide guidance to other medical schools in similar circumstances who need to plan and make suitable adjustments to clinical skills examinations under current pandemic conditions. The paper illustrates how it is possible to design and implement clinical skills examinations (OSCEs) to ensure the validity and reliability of high-stakes performance assessments whilst protecting the safety of all participants, minimising risk and maintaining defensibility to key stakeholders.

## Background

Singapore was one of the first countries to test, diagnose and treat people infected with COVID-19 in January 2020. On 7 February 2020, the Ministry of Health (MOH) in Singapore declared ‘DORSCON Orange’ (DORSCON refers to Disease Outbreak Response System Condition and is a risk assessment) ( MOH, 2020) prior to the WHO declaration of COVID-19 as a global pandemic on 11 March 2020.

Singapore has well-developed protocols since the 2003 outbreak of severe acute respiratory syndrome (SARS) and the H1N1 influenza 2009 pandemic ( MOH, 2019). One example is the practice of ‘cohorting’ or segregation which was activated early January 2020 for COVID-19, especially for healthcare teams. The healthcare workers are divided to work in ‘cohorts’ - small teams matched for essential skill sets, and everyone is advised to limit socialisation outside of the team. The logic is that if one health care worker falls ill with COVID-19, then only that team will be quarantined and the others can continue to provide care for patients. This concept is also extended to health care workers who are not patient-facing.

The immediate effect of DORSCON Level Orange was that all students in the three Singapore medical schools were no longer allowed to continue educational activities in clinical environments and there was advice from the MOH that gatherings of students should be limited to 50 people.

There was also a national level policy decision that medical students in their final year, who had completed all their clinical rotations and were 3 months away from graduation, should undertake their graduation level knowledge tests and clinical examinations. This was to avoid delaying graduation for current final year students and ensure those students who met the required standards could enter the workforce as newly qualified doctors, as house officers, an important addition to strained healthcare manpower resources.

We worked closely with the MOH and our healthcare partners to proceed with our final OSCE (Objective Structured Clinical Examination) ( Boursicot, Roberts *et al*., 2018): 25 stations, with community-based real and simulated patients, so that we could contribute more medical staff to the clinical working environment.

## Purpose

This paper is to document our experience of planning and conducting an OSCE during the COVID-19 pandemic, so that others in similar circumstances will be able to plan and make suitable adjustments to the clinical skills examinations that ensure the validity and reliability of such high-stakes assessments ( Lockyer, Carraccio *et al*., 2017) whilst protecting the safety of all participants, minimising risk and maintaining defensibility to key stakeholders.

The following key principles were applied throughout:

1. Strict infection control and personal hygiene; cleaning between circuits, face masks, hand sanitisation, health, travel declarations, temperature screening, solitary lunches
2. Cohorting or segregation of all participant groups including by patient, student, faculty and healthcare institution
3. Social distancing of individuals and physical isolation of different cohorts
4. Zoom-facilitated briefings
5. Wifi-enabled data gathering from iPad-based OSCE scoring system (already in place)
6. No large group gatherings

## Planning and delivery of the OSCE

Circuit design and allocation of students, examiners, real and simulated patients, and administrative staff

We divided students into four cohorts (14 students each, total number of students 56); the same cohorts were maintained throughout the three days of the examination and each cohort was managed separately, on a different circuit with different reporting and holding rooms. Each circuit was allocated specific administrative staff, and faculty did not cross over to other circuits.

The OSCE was conducted across a three day period and there were different examiners on each of the three days. Allocation to a circuit was according to hospital where an examiner usually worked; we avoided allocating examiners from different sites to the same circuit. At no time were students, staff or examiners from different circuits allowed to mix, and all were also given strict instructions about avoiding meeting and socializing after hours. This was to maintain the cohorting requirements of the healthcare institutions, as well as maintaining the student cohorts.

We were not permitted to use our usual clinical venue, which had been four parallel outpatient clinics, so we had to revise the plan to conduct the examinations in ‘non clinical buildings’. We used one medical school building, teaching rooms and clinical skills rooms, for two circuits. A non-clinical building owned by the main teaching hospital nearby was used for another two circuits: we used a floor of teaching rooms and set up beds and bedding, chairs, rest stations, and equipment for practical skills, to mirror the circuits in the medical school building. This enabled four parallel circuits.

All participant groups were cohorted. Students, examiners, external examiners, simulated patients (SPs) and community-based patients entered and left by different exits and circuits were conducted in a manner to ensure complete physical separation between circuits.

### General administrative arrangements

Administration on each of the three days of the examination involved

a. segregated participant registration areas at entrance of building
b. individual temperature taken on arrival, recorded, and displayed on sticker on clothing
c. individual travel declaration about last month of travel, any encounters with sick people
d. everyone wore masks, except for students and patients
e. hand sanitiser outside and inside every room
f. cohorts in different circuits directed to different toilets
g. no re-usable bedding, gowns, etc.
h. no non-essential touching (such as handshaking)
i. use of hand sanitiser before and after touching patients (real or simulated)
j. social distancing (one metre apart, except when examining patients)

### Special measures

Activities during the OSCE usually conducted in groups were adapted to ensure that there was minimal meeting between people involved in the examination

a. SPs kept in separate groups by circuit, staying in the room for their station all day
b. (real) patients kept in separate groups by circuit, staying in the room for their station all day. Dedicated nurses were provided for each cohort of patients
c. examiners allocated to one circuit, but not gathered or briefed together: after registration, they were escorted directly to their allocated room and examiners were not allowed out of the room until the end of the examination (apart from comfort breaks)
d. examiner briefing and calibration was conducted via Zoom on iPads which were also being used for scoring
e. students in groups of 14 maximum in a large room where they were required to sit at least one metre apart (for social distancing purposes)
f. no congregation of any people during coffee or lunch breaks: all food and drinks (individually packaged and sealed) were delivered and consumed in the individual room or examination room. There was no sharing of food or drinks; the open buffets originally planned were cancelled

### OSCE content

There were no changes made to the blueprint, station design and content, or standard setting procedures. As there was some difficulty in the recruitment of real patients, we had to replace some with simulated patients and the scoring rubric was adjusted accordingly in those stations (only two clinical examination stations).

The examination consisted of two parts:

1. The CPX (Clinical Performance Examination) consisted of 15 x 12 minute stations, including history taking, clinical examination and explanation/advice skills being tested, together with clinical decision making, diagnostic acumen and management planning.
2. The OSEPS (Objective Structured Examination of Practical Skills) consisted of 10 x 8 minute stations, mapped to the procedures dictated by the MOH document ‘Outcomes Framework’ 2018. These were ‘hybrid’ stations, where SPs were present in every station to appropriately situate the practical tasks, and engage the students in professional interactions with patients, including explanations, consent, and patient safety.

The scoring scheme was based on rating scales and rubrics, with some short checklists integrated for the technical elements of practical skills stations ( Wood and Pugh, 2020). The Borderline Regression Method ( Yousuf, Violato *et al*., 2015, Homer, Fuller *et al*., 2019) was used to set the passing scores. The scores of the CPX and OSEPS sections were not conjunctive.

## Challenges and solutions

### Healthcare sector

The MOH and directors of government health providers were very supportive and enabled us to:

• purchase bulk orders of hand sanitiser, masks, gloves (in an environment where suppliers were only allowed to sell to hospitals)
• obtain support for doctors to leave clinical work to be examiners

This official ministry level declared policy that it was a priority to proceed with the examination was important, as were clear ministry guidelines and written directives from senior management that clinicians coming for the examination would not flout ministry cohorting rules (since examiners from different hospitals were cohorted).

### Administrative

In a tight time frame of only four weeks we achieved a number of administration changes to implementation plans.

a. Restructuring of the whole system of circuit venues and creation of maps of direction of flow, highlighting separate entry and exits points of each building (to ensure physical separation of cohorts).
b. Reallocation of examiners to different circuits according to work site/hospital.
c. Recruitment of a new group of examiners to replace nurse educator examiners for the OSEPS part of the examination: all nurses were redeployed to front line patient care, and so could not examine in our OSCE. This necessitated the recruitment of 36 new clinician examiners at four weeks’ notice.
d. Telephone calls to reassure real patients and simulated patients about their safety, seek their consent to still participate, and recruit more where needed.
e. Examiner training/calibration sessions had been conducted face to face until DORSCON Orange level was declared. After that, the sessions were migrated into an online environment, with the use of videoconferencing (Zoom) for discussion.
f. Briefing at the start of each day conducted via Zoom, with each examiner at their respective OSCE station. Zoom enabled examiners on the same stations in the four parallel circuits to hold small group discussions for calibration.
g. All medical school staff leave was cancelled and many extra examiners were on standby in case of last minute absence on the day, an increased likelihood during the pandemic.
h. Extra SPs were recruited in the event of a no show by real patients.
i. Reconfirmation with external examiners (one from the UK, one from Australia, and one from Singapore) of intention to still attend the OSCE in Singapore.

### Students

We briefed the students during the month before the examinations were scheduled.

Students were also keen to avoid delay with graduation and were already mentally prepared for examinations. During the briefing to students, we included a senior Infectious Disease consultant, the Dean of the medical school, Clinical Deans, and the Vice Dean for Education. Senior clinicians were present, all of whom were continuing to work clinically.

### Patients

A major concern was with bringing actual patients back for the examination. This was a multipronged pronged challenge - firstly, we were not permitted to use any inpatients or patients with any healthcare contact in the preceding 2 weeks. Secondly, there was initially fear amongst the patients about coming back to a healthcare facility as there was a perception that this might increase the risk of acquiring the COVID infection. Thirdly, there was of course the risk that one of the patients might actually have contracted and been a spreader of the disease. On the other hand, we felt that not being able to test the students’ ability to pick up physical signs would impact the validity of the examination.

We were able to overcome this by utilizing a pool of very stable patients with chronic conditions, minimising the exchange of patients during the examination, and by rigorous screening of patients prior to attending for the examination. All patients had to fulfil these requirements:

• no travel history or any sick family members in the two weeks prior to the examination.
• patients were telephoned to check on this twice in the two weeks prior to the examination.

Patients were reassured that the examination was to be held at a location separate from the hospital, that the other people involved in the examination were to be thoroughly screened and that patient safety precautions would be paramount.

## Post-OSCE reflection and conclusion

To date, there have been no reports of COVID-19 infections, by those who participated in the OSCE. External examiners provided reports that commended the measures adopted and affirmed the defensibility of the examination results. The students who failed the OSCE will have a supplementary exam after two months and will have another opportunity to demonstrate their readiness for clinical practice.

In a time of outbreak, the primary concern is, and should be for the patients; training needs necessarily take a back seat. And yet it is still our duty to train the next generation of doctors. Our planning and adaptation to COVID-19 conditions reflects the values of the medical profession and the importance we place on students being prepared for the clinical workplace. By proceeding with the examination, we believe that we have sent a powerful signal to our nascent young doctors that in their chosen profession, we stay calm and carry on with important work, while taking appropriate precautions to ensure everyone’s safety.

## Take Home Messages

• It is possible to take sufficient measures in a pandemic environment to conduct clinical examinations safely.
• These measures require the collective support of the healthcare sector, medical school academic and administrative staff, students, examiners and external examiners, simulated patients and patients.
• Good communication and collaborative working are key factors.
• Think imaginatively, be flexible and nimble.

## Notes On Contributors

**Katharine Boursicot** is the Associate Dean for Assessment and Progression, Duke-National University of Singapore Medical School, Singapore.

Medical educationalist, overall academic director of OSCEs at Duke-NUS, lead for blueprinting, design of stations, standard setting, examiner training.

Main author, conceptualized, wrote and revised manuscript based on comments and suggestions from the other authors.

**Sandra Kemp**, Professor of Medical Education, is the Director, Learning and Teaching, Curtin Medical School, Curtin University, Perth, Australia.

An external examiner for the OSCE. Contributed to the conceptualization of the paper, reviewed and revised drafts.

**Thun How Ong**, Singapore General Hospital.

Senior respiratory physician, Clinical Lead for Assessment at Duke-NUS, clinical lead for the OSCE, worked to recruit more examiners, ensure minimisation of cross-infection, delivered on line examiner briefing and calibration.

Reviewed the early drafts, with fact checking and suggestions for improvement.

**Limin Wijaya**, Singapore General Hospital.

Senior infectious diseases physician, Co-lead for OSCE, advised on infection control measures.

Reviewed drafts, advised on the correct national level directives.

**Sok Hong Goh**, Duke-National University of Singapore Medical School, Singapore.

Senior Manager, Office of Education.

*Overall administrative lead, sourced required infection control supplies, new venue, cohorting of administrative staff.*

Reviewed drafts, fact checked information about practical arrangements.

**Kirsty Freeman**, Duke-National University of Singapore Medical School, Singapore.

Lead Associate for Simulation,Worked to revise circuit plans of OSCE, cohorting arrangements, ensured supplies of adequate equipment in new venue, supervised 2 OSCE circuits.

Reviewed draft.

**Ian Curran**, Vice-Dean for Education, Duke-National University of Singapore Medical School, Singapore.

Reviewed drafts, advised on national level and institutional directives.

## References

[ref1] Boursicot K. A. M. Roberts T. E. and Burdick W. P. (2018). Structured Assessments of Clinical Competence. Understanding Medical Education. London, Wiley:335–345. 10.1002/9781119373780.ch23

[ref2] Homer M. Fuller R. Hallam J. and Pell G. (2019) Setting defensible standards in small cohort OSCEs: Understanding better when borderline regression can ‘work’. Medical Teacher. 1–10. 10.1080/0142159X.2019.1681388 31657266

[ref3] Lockyer J. Carraccio C. Chan M. K. Hart D. (2017) Core principles of assessment in competency-based medical education. Medical Teacher. 39(6):609–616. 10.1080/0142159X.2017.1315082 28598746

[ref4] MOH (2019). Being prepapred for a pandemic. Singapore MOH website. https://www.moh.gov.sg/diseases-updates/being-prepared-for-a-pandemic( Accessed: 23 March 2020).

[ref5] MOH (2020). Risk assessment raised to DORSCON Orange. Singapore MOH website. https://www.gov.sg/article/what-do-the-different-dorscon-levels-mean( Accessed: 23 March 2020).

[ref6] Wood T. J. and Pugh D. (2020) Are rating scales really better than checklists for measuring increasing levels of expertise? Medical Teacher. 42(1):46–51. 10.1080/0142159X.2019.1652260 31429366

[ref7] Yousuf N. Violato C. and Zuberi R. W. (2015). Standard Setting Methods for Pass/Fail Decisions on High-Stakes Objective Structured Clinical Examinations: A Validity Study. Teaching and Learning in Medicine. 27(3):280–291. 10.1080/10401334.2015.1044749 26158330

