# Supplementary material for: Conducting a high-stakes OSCE in a COVID-19 environment
Source: MedEdPublish (2016). 2020 Mar 27;9:54. [Version 1] doi: 10.15694/mep.2020.000054.1 (PMC10697458; doi:10.15694/mep.2020.000054.1)
Supplement: Peer review history

# Reviewer report: Reviewer response for version 1

**DOI:** 10.21956/mep.20025.r30579

**Published:** 2020-04-03

**Authors:** Shaoting Feng

**Affiliations:** 1 The First Affiliated Hospital of Sun Yat-sen University

This review has been migrated. The reviewer awarded 5 stars out of 5 This paper provides us practical guidance in details to plan and make suitable adjustments to clinical skills examinations under current pandemic conditions. It's very enlightening! Thanks for sharing!

Reviewer Expertise:

NA

No decision status is available

# Reviewer report: Reviewer response for version 1

**DOI:** 10.21956/mep.20025.r30577

**Published:** 2020-04-02

**Authors:** Richard Hays

**Affiliations:** 1 James Cook University

This review has been migrated. The reviewer awarded 5 stars out of 5 This is a timely paper and provides a comprehensive approach to running an OSCE with all participants on-site. The issue not addressed, but not intended to be addressed, is how can a high-stakes OSCE be managed with physical separation between some participants? It could be argued that moving people around and between health facilities is currently unwise. One model to consider is that of the Australian College of Rural and Remote Medicine (ACRRM), which offers multi-station clinical examinations remotely, because rural docs find it difficult to leave their communities (see: Smith J, Prideaux D, Wolfe CL, Wilkinson TJ, Sen Gupta T, DeWitt D, Worley P, Hays RB, Cowie M. Developing the accredited postgraduate assessment program for Fellowship of the Australian College of Rural and Remote Medicine. Rural and Remote Health 2007; 7: 805. Available: www.rrh.org.au/journal/article/805). Th ACRRM model is smaller scale and has simulated patients in a different location, to candidates and examiners but is much smaller scale than an OSCE in a large medical school. However, it seems to work. Would it be possible to have students and (real or simulated) patients at one site, moving around as usual, with examiners in another location watching by video? This would substantially reduce the number of clinicians gathering and interacting with students. If simulated patients (including people with stable signs not currently unwell) were used, the impact on a health care facility should be reduced. Something like this might be worth considering as tighter social distancing is introduced.

Reviewer Expertise:

NA

No decision status is available

# Reviewer report: Reviewer response for version 1

**DOI:** 10.21956/mep.20025.r30581

**Published:** 2020-03-31

**Authors:** Tripti Srivastava

**Affiliations:** 1 Datta Meghe Institute of Medical Sciences Deemed University

This review has been migrated. The reviewer awarded 2 stars out of 5 An innovative approach to conduct assessments in challenging situations. weighing the nature of assessment against the perceived risk factors is the most critical aspect of undertaking such initiative. Authors have tried to share their experience of one such initiative focussed on minimising the risk for students , assessors and patients. This case study can be a guide for conducting such assessments, without going into the scientific details of the study.

Reviewer Expertise:

NA

No decision status is available

# Reviewer report: Reviewer response for version 1

**DOI:** 10.21956/mep.20025.r30582

**Published:** 2020-03-29

**Authors:** Wendy Hu

**Affiliations:** 1 School of Medicine, Western Sydney University

This review has been migrated. The reviewer awarded 5 stars out of 5 I congratulate the authors in rapid publication of this extremely timely information, which is of great interest to educators worldwide in these "testing times". A great strength of the report is the level of contextual detai provided, allowing readers to take what is workable in their situation. While educational evaluation is yet to come, the process quality assurance, relationship to evidence, and vitally, the absence of infection in any participants, is key. The unusual situation created a new purpose - to ensure infection control to the highest standard, and the procedures appear to have met this aim. The altenrnative of suspending live clinical examinations is however, being faced by many medical schools and I wonder if the authors would like to explore this option in future publications? The Singapore success in COVID control has been globally recognised, and the medical school's success is testament of the deep relationships between health care, government and educational systems, which the authors acknowledge as a strength. However, for other contexts, this may be lacking, and a relative lack of resources will preclude the measures described. Authors in less well resourced contexts, should also be encouraged to contribute their experiences and not see this example as best practice that they must emulate. The publication is also testament to the flexibility of the MedEdPublish format and its fit with practitioners and innovation focussed researchers' information needs. If anything is to be gained from the current crisis, it is the rapid and huge increase in research and release of results based on "live" data seen in all major medical journals in recent months, and to be emulated by medical and heatlh professions education research. given our interdependency and integration with clinical practice.

Reviewer Expertise:

NA

No decision status is available

# Reviewer report: Reviewer response for version 1

**DOI:** 10.21956/mep.20025.r30583

**Published:** 2020-03-29

**Authors:** P Ravi Shankar

**Affiliations:** 1 American International Medical University

This review has been migrated. The reviewer awarded 5 stars out of 5 The authors describe conducting a high-stakes OSCE during the pandemic. Though it has not been explicitly stated, I assume this is the final clinical exam for students prior to graduation. The authors have described the logistics and preparation very well. The use of Zoom for briefing and for examiners to communicate with each other is interesting. The use of iPad for electronic contact and scoring minimized physical contact. The modality approached for separating students, examiners and patients has been highlighted. The separate packaging of food and refreshments and delivery of food to individual rooms has been mentioned. Methods to minimize physical contact have been well described. I have a question about the external examiners. What was their exact role? Were they physically present or did they participate electronically? The class size is relatively small and I am thinking about possible challenges in scaling up the process to class sizes of 200 to 250 students. The initiative will be of special interest to educators in these challenging times though adaptations to local circumstances may be required.

Reviewer Expertise:

NA

No decision status is available

# Reviewer report: Reviewer response for version 1

**DOI:** 10.21956/mep.20025.r30580

**Published:** 2020-03-27

**Authors:** Judy McKimm

**Affiliations:** 1 Swansea University

This review has been migrated. The reviewer awarded 5 stars out of 5 A very timely and clear description of how to be adaptive in times of rapid change. I liked the discussion of how you worked to allay patients' fears and the care taken to minimise cross infection etc. The timings are crucial aren't they for what is possible, and clear communications to everyone, plus contingency planning are vital. It would be very interesting to gather ideas about how others have tackled the practical/clinical skills assessment in these times.

Reviewer Expertise:

NA

No decision status is available

# Reviewer report: Reviewer response for version 1

**DOI:** 10.21956/mep.20025.r30576

**Published:** 2020-03-27

**Authors:** Trudie Roberts

**Affiliations:** 1 Leeds Institute of Medical Education

This review has been migrated. The reviewer awarded 5 stars out of 5 Another valuable contribution to the stable of articles on managing educational activities or 'Learning in a time of Covid' to paraphrase Gabriel Garcia Marquez. This event ran extremely well - I know I was one of the external examiners - but only because of the commitment of the clinicians, academics, administrator, patients - real and simulated and students. As the authors comment thinking imaginatively and flexibly, looking at the opportunities that newer technologies is one of the keys to success in these challenging times. Congratulations to these educators on this achievement.

Reviewer Expertise:

NA

No decision status is available

# Reviewer report: Reviewer response for version 1

**DOI:** 10.21956/mep.20025.r30578

**Published:** 2020-03-27

**Authors:** Deb Halder

This review has been migrated. The reviewer awarded 5 stars out of 5 Review on the PaperThe Crucial Points to focus on:i. The title of the paper uses ‘high-stakes’ though it has never been clarified why ‘OSCE’ is ‘high-stakes’. ii. An introduction is inseparable part for a case study where the considered issues are focused, the title is analyzed and the area of study is specified that the paper deliberately passes over.iii. In the background there is an excerpt that ‘MOH advises gatherings of 50 clinical students at a time and place’. But, this remark has not been proceeded with any citation which makes this statement little valid and credible. iv. Case study develops with lots of references along with the prevailing case. But, this paper is found to have used less related references to defend why these measures should be taken, what is advised by WHO and what they need to follow. These are few points that knocks the criteria of credibility of actions. Few Syntactic Issues: Few grammatical, lexical issues attract attention. For example, the very first sentence of the abstract uses ‘presented’ as the main verb of the paper which should be ‘posed’ or ‘created’.The very two lines under planning and delivery of OSCE tends to be sound with syntactical harmony. The Strongest Part of the Paper:The Case study is presented in details. An order has been followed to designate the adopted measures point by point so that no important issue is missed. This detailed description of ways and procedures make these ways adoptable.

Reviewer Expertise:

NA

No decision status is available

# Reviewer report: Reviewer response for version 1

**DOI:** 10.21956/mep.20025.r30575

**Published:** 2020-03-27

**Authors:** Trevor Gibbs

**Affiliations:** 1 AMEE

This review has been migrated. The reviewer awarded 5 stars out of 5 A very insightful and appropriate paper published at exactly the right time. As we all continue to worry and develop newer approaches to standardised methods, this paper clearly shows that with thought, that we can continue with a tried and tested assessment approach.I will look forward to hearing the student / patient and faculty evaluations of this activity went, but I hve ne reservations in recommending this paper to all curriculum developers

Reviewer Expertise:

NA

No decision status is available

# Reviewer report: Reviewer response for version 1

**DOI:** 10.21956/mep.20025.r30574

**Published:** 2020-03-27

**Authors:** Felix Silwimba

**Affiliations:** 1 University of Lusaka

This review has been migrated. The reviewer awarded 5 stars out of 5 very informative and informative article on medical education in times of a pandemic. it definitely addresses concerns of all medical schools world over

Reviewer Expertise:

NA

No decision status is available
